# Salidroside Improves Oocyte Competence of Reproductively Old Mice by Enhancing Mitophagy

**DOI:** 10.1111/acel.14475

**Published:** 2025-01-09

**Authors:** Jingkai Gu, Renwu Hua, Huayan Wu, Chenxi Guo, Zhuo Hai, Yuan Xiao, William S. B. Yeung, Kui Liu, Elnur Babayev, Tianren Wang

**Affiliations:** ^1^ The University of Hong Kong‐Shenzhen Hospital Shenzhen Guangdong China; ^2^ Department of Obstetrics and Gynaecology, Li Ka Shing Faculty of Medicine The University of Hong Kong Hong Kong SAR China; ^3^ Department of Obstetrics and Gynecology, Feinberg School of Medicine Northwestern University Chicago Illinois USA

**Keywords:** aging, ART, egg, IVF, mitochondria, ovary

## Abstract

The decline of oocyte quality with advanced maternal age has a detrimental effect on female fertility. However, there is limited knowledge of therapeutic options and their mechanisms to improve oocyte quality in reproductively older women. In this study, we demonstrated that supplementation of salidroside improves the oocyte quality of reproductively old mice. Salidroside improved the maturation, fertilization, and developmental competence of oocytes from reproductively old mice by maintaining the normal spindle/chromosome structure and mitochondrial function. Oocyte transcriptomic and micro‐proteomic analysis revealed that salidroside restores oocyte quality by enhancing mitophagy in reproductively old mice. Our studies provide a new theoretical foundation for utilizing salidroside to improve oocyte quality in reproductively old females in the context of natural fertility or assisted reproduction.

## Graphical abstract

Salidroside improves oocyte competence by enhancing mitophagy. Aging impairs mitophagy in oocytes, which leads to mitochondrial dysfunction. Salidroside restores mitochondrial homeostasis, reduces ROS levels, and improves oocyte developmental competence.

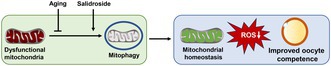

## 1 Introduction

Ovarian reproductive function starts to decline after the early 30s, and this trend becomes more dramatic after the age of 37 (Dong et al. 2023; Faddy et al. 1992; Heffner 2004; te Velde and Pearson 2002). Ovarian aging is characterized by decreasing follicle numbers and oocyte quality (te Velde and Pearson 2002; Yamamoto et al. 2010). Delayed childbearing in the modern world has led to the increasing infertility rates. Assisted reproductive technologies (ART) cannot overcome the reproductive decline women face with advanced maternal age (Seshadri et al. 2021). Reproductive aging also has implications for the women's overall health, with increased rates of osteoporosis, cardiovascular disease, and neurological dysfunction after menopause (Ahanchi et al. 2024; Scanferla et al. 1987).

Previous studies have shown that optimal mitochondria function and genes involved in mitochondrial quality control, such as *Clpp*, *Mfn1*, *Mfn2*, and *Lonp1*, are essential for oocyte competence, and regulate ovarian aging (Babayev and Seli 2015; Babayev et al. 2016; Carvalho et al. 2020; Sheng et al. 2022; T. Wang et al. 2018a; M. Zhang et al. 2019). An increasing number of studies have reported that various supplements, including nicotinamide mononucleotide, spermidine, Coenzyme Q10, MitoQ, BGP‐15, C‐type natriuretic peptide, quercetin, and N‐acetyl‐l‐cysteine, can enhance folliculogenesis and/or oocyte quality by improving mitochondrial function; however, the data are limited (Al‐Zubaidi et al. 2021; Ben‐Meir et al. 2015; Cao et al. 2020; J. Liu et al. 2012; Y. Miao et al. 2020; H. Zhang et al. 2023b; Y. Zhang et al. 2023a). To date, there are no approved drugs that target ovarian aging. Therefore, there is an urgent need to understand the mechanisms of reproductive aging and develop mitigating and therapeutic strategies.

Salidroside [2‐(4‐Hydroxyphenyl)ethyl‐β‐d‐glucopyranoside] is one of the main active ingredients in the root of *Rhodiola rosea*, which is a traditional Chinese medicine with low toxicity and few side effects (J. Zhang et al. 2023c; X. Zhang et al. 2021). Salidroside has been regarded as an effective medicine to prevent and treat high‐altitude illness (Qian, Ge, and Kong 2012). Recent studies demonstrated that it also has a variety of pharmacological actions, including anti‐aging (Cai et al. 2021; Mao et al. 2015; S. Wang et al. 2015; X. Wang et al. 2018b), antioxidation (Chen et al. 2009), anti‐inflammatory (Guan et al. 2012), and antiviral (H. Wang et al. 2009) properties. Salidroside supplements have been demonstrated to extend the lifespan of the fish *Nothobranchius guentheri* (X. Wang et al. 2018b), reverse the premature senescence of human skin fibroblasts (Mao et al. 2015), and have beneficial effects in Alzheimer's and Parkinson disease (Cai et al. 2021; S. Wang et al. 2015). Mechanistically, salidroside appears to regulate IGF, mTOR, AMPK, and Sirtuins to modulate aging (Amorim et al. 2022; X. Zhang et al. 2021).

The ovary is one of the first organs to age (Wu et al. 2022). A recent study has reported that salidroside increases the oocyte maturation and blastocyst formation rate in pigs (Shi et al. 2023). However, it is not known how salidroside affects ovarian aging. In this study, we tested the hypothesis that salidroside ameliorates reproductive aging. Salidroside was supplemented in vivo by intraperitoneal injection into reproductively old mice or in vitro by addition to the oocyte culture medium. We discovered that salidroside supplementation improves the fertility and oocyte competence of reproductively old mice. Salidroside improved mitochondrial membrane potential, and ATP and reactive oxygen species (ROS) levels in reproductively old mice oocytes. Our transcriptomic and proteomic analysis revealed that the regulation of mitophagy appears to be the main mechanism of salidroside action in mouse oocytes. This study provides important insights into the therapeutic role of salidroside in ovarian aging and paves the way for translational studies in humans.

## 2 Materials and Methods

An expanded section describing materials and methods is available in Supporting Information Data S1.

## 3 Results

### 3.1 Salidroside Improves Folliculogenesis, Oocyte Developmental Competence, and Fertility of Aging Mice

To examine salidroside pharmacokinetics, we analyzed the serum concentration of salidroside in 8‐week‐old C57BL/6 female mice after 50 mg/kg intraperitoneal salidroside injection. Plasma concentration of salidroside reached 41,392 ± 8247 ng/mL at 0.25 h and decreased to 216 ± 115 ng/mL at 4 h, suggesting that salidroside is rapidly absorbed and cleared after intraperitoneal administration (Figure S1).

**FIGURE 1:**
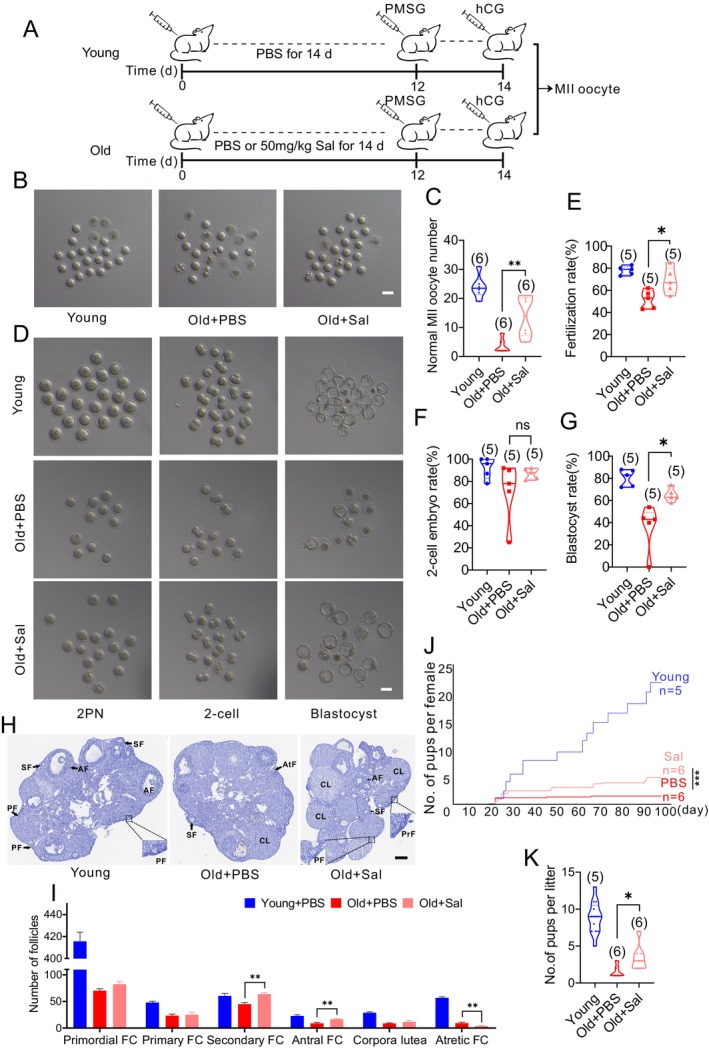
Salidroside (Sal) treatment improves the fertility of reproductively old (10 months) female mice. (A) Timeline diagram for Sal administration, hormone priming, and subsequent analyses. GV, germinal vesicle; hCG, human chorionic gonadotropin; MII, metaphase II; PMSG, pregnant mare serum gonadotropin. (B, C) Representative images of ovulated oocytes after 14 days of Sal or PBS treatment and corresponding analysis. Scale bar: 80 μm. (D) Representative images of embryos at various developmental stages following in vitro fertilization. Scale bar: 80 μm. (E–G) Fertilization, two‐cell embryo development, and blastocyst development rates after in vitro fertilization. Total number of oocytes used were: Young (*n* = 110), Old + PBS (*n* = 59), and Old + Sal (*n* = 82) from five independent experiments (H). Representative images of ovarian morphology across groups. Scale bar: 320 μm. AF, antral follicle; AtF, atretic follicle; CL, corpus luteum; PF, primary follicle; PrF, primordial follicle; SF, secondary follicle. (I) Ovarian follicle counts in young (*n* = 3), old (*n* = 5), and Sal + old (*n* = 5) mice FC: Follicle (J) Breeding trials demonstrate the total number of pups per female (K) and litter size (*n* = 6) over time for 14 weeks after mating with young male mice. ns: *P* > 0.05; *: *P* < 0.05; **: *P* < 0.01; and ***: *P* < 0.001. The bar graph displays the mean ± SEM of the results obtained from at least three mice. The truncated violin plots depict the median, quantiles and individual data points. Numbers in parentheses represent the number of mice. One‐way ANOVA was used for statistical analysis.

To further investigate the effect of salidroside on oocytes from reproductively old (10‐month‐old) mice, we administered salidroside or PBS via intraperitoneal injection for a duration of 14 days (Figure 1A) (Cao et al. 2020; Nemerovsky et al. 2022). After superovulation, the number of total ovulated oocytes obtained in the treatment group was not different from old controls (Figure 1B); however, the quantity of healthy‐looking, nondegenerating MII oocytes was significantly higher in the salidroside injection group (Figure 1C). To assess the developmental potential of oocytes following salidroside treatment, we performed IVF (Figure 1D). Salidroside improved fertilization (Figure 1E) and blastocyst development rate (Figure 1G) in reproductively old mice. There was no difference in two‐cell embryo development rate across groups (Figure 1F). Salidroside treatment led to the increase in the number of secondary and antral follicles, and to a concurrent reduction of atretic follicles (Figure 1H,I). Salidroside also increased the number of pups per litter and the number of total pups per female in the reproductively old group (Figure 1J,K). Collectively, these data suggest that salidroside improves folliculogenesis and oocyte developmental capacity in reproductively old mice.

### 3.2 Salidroside Improves Germinal Vesicle Oocyte Antioxidant Capacity and Mitochondrial Function In Vivo

**FIGURE 2:**
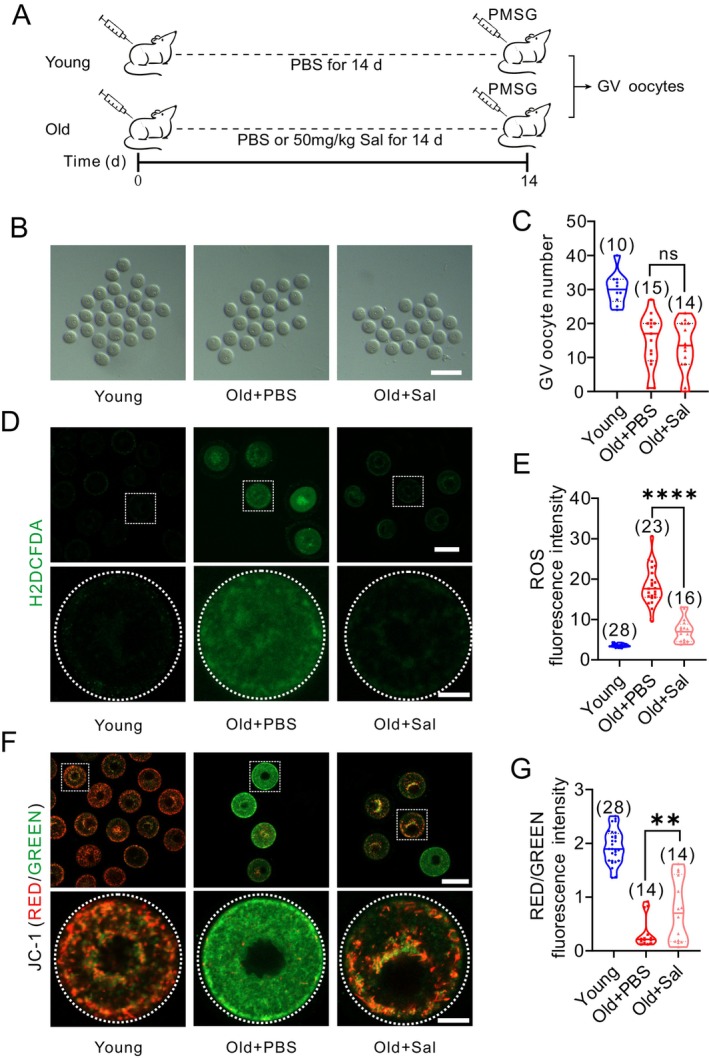
Salidroside (Sal) improves antioxidant capacity and mitochondrial function in germinal vesicle stage oocytes in reproductively old mice. (A) Timeline diagram for Sal administration, hormone priming, and subsequent analyses. GV: germinal vesicle; hCG, human chorionic gonadotropin; MII, metaphase II; PMSG, pregnant mare serum gonadotropin. (B) Representative images of GV oocytes from Young, Old + PBS, and Old+ Sal mice. Scale bar: 160 μm. (C) The number of GV oocytes obtained after PMSG injection. (D) ROS levels in Young, Old + PBS, and Old + Sal mice GV oocytes after H_2_O_2_ exposure as detected by Carboxy‐H2DCFDA, scale bar: 80 and 20 μm, (E) and quantification of ROS fluorescence intensity. (F) Representative images and quantification (G) of mitochondrial membrane potential (ΔΨm) as assessed by JC‐1 staining in GV oocytes from Young, Old + PBS, and Old + Sal mice (red, high ΔΨm; green, low ΔΨm). Scale bar: 80 and 20 μm. ns: *P* > 0.05; **: *P* < 0.01; ****: *P* < 0.0001. The truncated violin plots depict the median, quantiles and individual data points. Numbers in parentheses represent the number of mice for (C) and number of oocytes for (E) and (G). One‐way ANOVA was used for statistical analysis.

We tested the effects of salidroside supplementation on mitochondrial function in aged oocytes. The number of GV oocytes did not significantly change after superovulation following salidroside treatment in reproductively old mice. (Figure 2A–C). However, salidroside treatment reduced ROS levels in reproductively old mice GV oocytes (Figure 2D,E). Salidroside also significantly improved the mitochondrial membrane potential of reproductively old mice GV oocytes, as evidenced by increased red fluorescence in JC‐1 reagent‐based assay (Figure 2F,G).

### 3.3 Salidroside Improves Maturation and Enhances the Mitochondrial Function of Oocytes In Vitro

**FIGURE 3:**
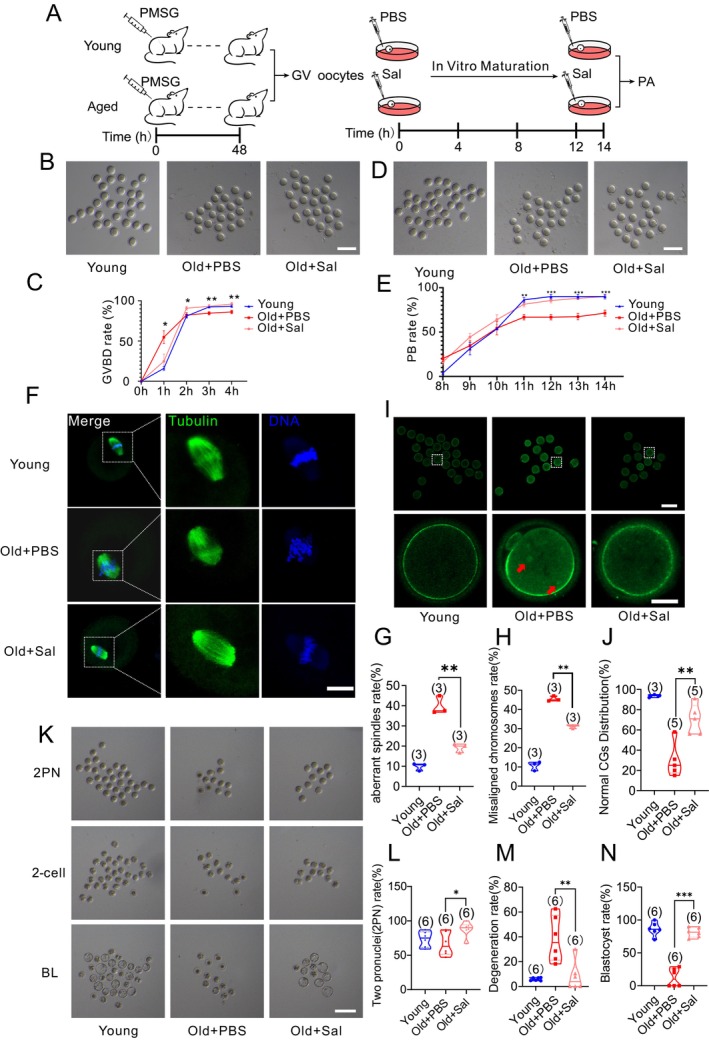
Salidroside (Sal) improves oocyte in vitro maturation (IVM) in reproductively old mice. (A) Schematic diagram of in vitro treatment of aged mice oocytes with Sal. GV, germinal vesicle; PA, parthenogenetic activation; PMSG, pregnant mare serum gonadotropin. (B) Representative images and (C) quantification of germinal vesicle breakdown (GVBD) of oocytes from Young (*n* = 3), Old + PBS (*n* = 5), and Old + Sal (*n* = 5) mice after 4 h of IVM. Scale bar: 160 μm. (D) Representative images of oocyte maturation and (E) quantification of polar body (PB) extrusion rate from Young (*n* = 3), Old + PBS (*n* = 5), and Old + Sal (*n* = 5) mice after IVM. Scale bar: 160 μm. (F) Representative oocyte spindle and chromosome morphology images from Young, Old + PBS, and Old + Sal treatment. Scale bar: 12.5 μm. (G) The percentage of aberrant spindles was quantified in oocytes from Young (*n* = 81), Old + PBS (*n* = 55), and Old + Sal (*n* = 57) treatment. (H) The percentage of misaligned chromosomes was quantified in oocytes from Young (*n* = 75), Old+ PBS (*n* = 55), and Old + Sal (*n* = 64) treatment. (I) Representative images of cortical granules (CGs) distribution in oocytes from Young, Old + PBS, and Old + Sal mice. Scale bar: 80 and 25 μm. Red arrows: Cortical granules that cannot attach properly to the cell membrane. (J) Normal CG distribution rate in metaphase II oocytes from Young (*n* = 62), Old + PBS (*n* = 97), and Old + Sal (*n* = 77) mice. (K) Representative images of parthenogenetically activated oocytes at various stages of development after treatment with Sal. Scale bar: 250 μm. (L) 2PN parthenogenetic activation (M) degeneration and (N) blastocyst development rate of Young (*n* = 110), Old + PBS (*n* = 59), and Old + Sal (*n* = 82) mice oocytes. *: *P* < 0.05; **: *P* < 0.01; ***: *P* < 0.001. The line graphs display the mean ± SEM of the results obtained from at least three mice. The truncated violin plots depict the median, quantiles and individual data points. Numbers in parentheses represent the number of mice. One‐way ANOVA was used for statistical analysis.

We investigated the impacts of in vitro salidroside supplementation on oocyte quality (Figure 3A). Salidroside improved germinal vesicle breakdown (GVBD) (Figure 3B,C) and expulsion of the first polar body (PB) (Figure 3D,E) of GV oocytes from reproductively old mice. Salidroside‐treated reproductively old mice demonstrated improved spindle morphology and reduced number of mature oocytes with misaligned chromosomes (Figure 3F–H). Additionally, salidroside ensured aggregation of cortical granules under oolemma similar to reproductively young mice (Figure 3I,J). This indicates that salidroside may also have an effect on oocyte cytoplasmic maturation. Given the low efficiency of conventional IVF in denuded (i.e., cumulus‐cell free) oocytes (Kimura and Yanagimachi 1995) and to exclude the impact of the introduction of sperm genome on embryonic development, we used parthenogenetic activation to test the developmental competence of the resultant mature oocytes (Figure 3K). Salidroside improved 2PN and blastocyst development rate and reduced embryo degeneration (Figure 3L–N).

**FIGURE 4:**
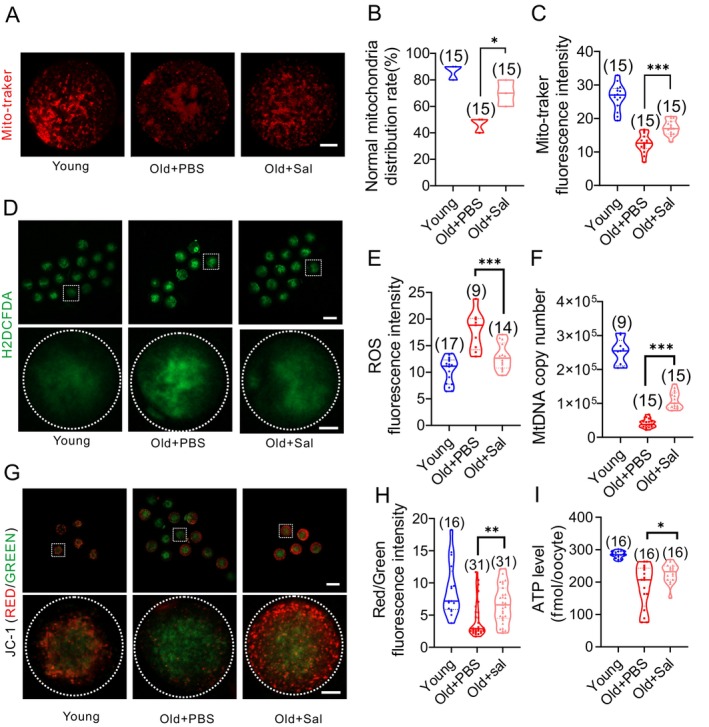
Salidroside (Sal) enhances oocyte mitochondrial function during in vitro maturation (IVM) (A) Representative images of mito‐tracker staining of MII oocytes after IVM. Scale bar: 20 μm. (B) The normal mitochondria distribution rate and (C) The fluorescence intensity of mitochondria in mature oocytes from reproductively old mice after Sal treatment. (D) ROS levels in Young, Old + PBS, and Old + Sal mice mature oocytes after H_2_O_2_ exposure as detected by Carboxy‐H2DCFDA, scale bar: 80 and 20 μm, (E) and quantification of ROS fluorescence intensity. (F) mtDNA copy number in Young, Old + PBS, and Old + Sal MII oocytes. (G) Representative images and (H) quantification of mitochondrial membrane potential (ΔΨm) as assessed by JC‐1 staining in MII oocytes, scale bar: 80 and 20 μm. (I) ATP levels in Young, Old + PBS, and Old + Sal MII oocytes. *: *P* < 0.05; **: *P* < 0.01; ***: *P* < 0.001. The truncated violin plots depict the median, quantiles and individual data points. Numbers in parentheses represent the number of oocytes. One‐way ANOVA was used for statistical analysis.

Salidroside treatment during IVM decreased the number of reproductively old mice oocytes with irregularly aggregated mitochondria (Figure 4A,B). The MitoTracker Red fluorescence intensity was increased after salidroside treatment indicating improved mitochondrial biogenesis (Figure 4A,C). Transcript levels of *Opa1*, *Mfn1*, *Mff*, and *Fis1* were also significantly increased after the treatment suggesting improved mitochondrial dynamics (Figure S2). Moreover, salidroside reduced ROS levels (Figure 4D,E), improved ATP levels (Figure 4I), and mitochondrial membrane potential (Figure 4G,H), and increased mitochondrial DNA number in aged mice oocytes (Figure 4F).

### 3.4 Transcriptomic and Micro‐Proteomic Analysis Reveal Autophagy as One of the Top Pathways Modulated in Aged Mouse Oocytes With Salidroside Treatment

**FIGURE 5:**
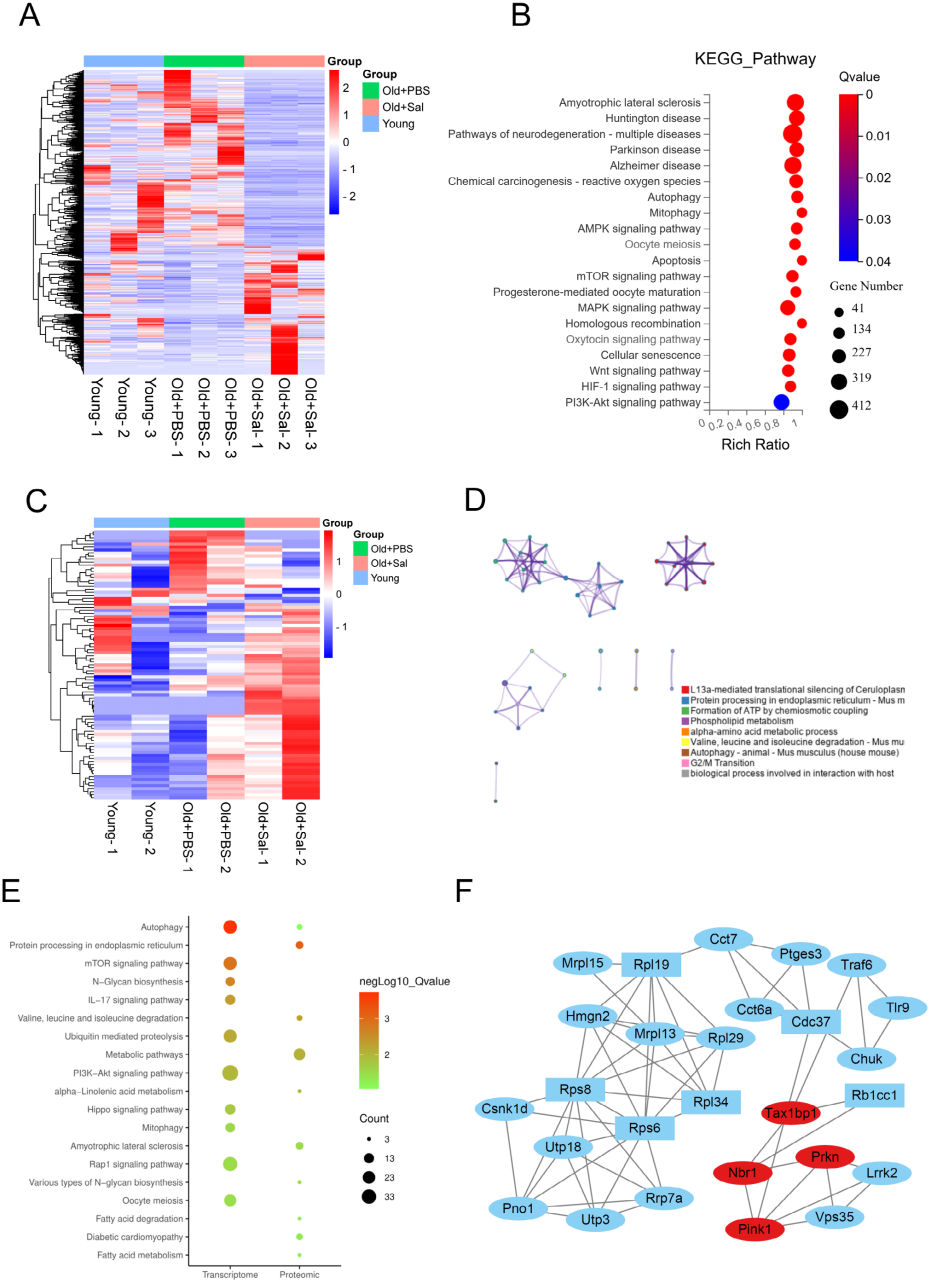
Transcriptomic and micro‐proteomic analysis of oocytes reveal pathways modulated by Sal. Transcriptomic analysis: (A) Heatmap and unsupervised hierarchical clustering of gene expression in Young, Old + PBS, and Old + Sal GV oocytes. (B) KEGG enrichment analysis of differentially expressed genes in Old + Sal compared to Old + PBS GV oocytes. Micro‐proteomic analysis: (C) Heatmap displaying protein expression in Old + PBS and Old + Sal GV oocytes. (D) Metascape analysis of differentially expressed proteins in Old + Sal compared to Old + PBS GV oocytes. Multi‐omics analysis: (E) KEGG pathway co‐enrichment analysis of differentially expressed genes and proteins in Old + PBS and Old + Sal GV oocytes. (F) Differentially expressed protein‐gene interaction regulatory networks. Rectangles and ovals represent differentially expressed genes and proteins, respectively. Red indicates genes related to mitochondrial autophagy function.

To dissect the mechanisms by which salidroside improves the quality of oocytes in reproductively old mice, we conducted transcriptomic and micro‐proteomic analyses of oocytes after 14 days of in vivo treatment. Heat map analysis of the transcriptomes demonstrated that salidroside partially restored gene expression changes in reproductively old mice GV oocytes (Figure 5A). Further analysis revealed that 388 genes were upregulated and 534 were downregulated in the salidroside treatment group compared to control reproductively old mice (Table S2). A total of 3296 genes were significantly differentially expressed in MII oocyte comparison groups (180 upregulated and 3116 downregulated, Figure S3A, Table S3). Among these differentially expressed genes, 202 genes were overlapping between GV and MII groups (Figure S3B). Autophagy and mitophagy were among the top enriched pathways of differentially expressed genes in both GV and MII oocyte comparison groups (Figures 5B and S3C). Similarly, GO enrichment analysis showed that autophagy and macroautophagy were enriched in MII oocytes comparison groups (Figure S3D).

Micro‐proteomic analysis of GV oocytes demonstrated that salidroside treatment changed the levels of 89 proteins: 63 proteins exhibiting upregulation and 26 proteins displaying downregulation (Figure 5C, Table S4). Autophagy was again among the top altered pathways (Figure 5D).

Through multiomics association analysis of the transcriptome and proteome, we found that autophagy was the top enriched pathway in both transcriptomic and microproteomic analyses of GV oocytes (Figure 5E). The interaction network of differentially expressed genes and proteins indicated that mitophagy‐related genes, including *Tax1bp1*, *Nbr1*, *Pink1*, and *Prkn*, were present in the Cluster1 network (Figure 5F). These data suggest that salidroside enhances oocyte competence at least in part by mitophagy regulation.

### 3.5 Salidroside Improves Mitophagy in Oocytes From Reproductively Old Mice

**FIGURE 6:**
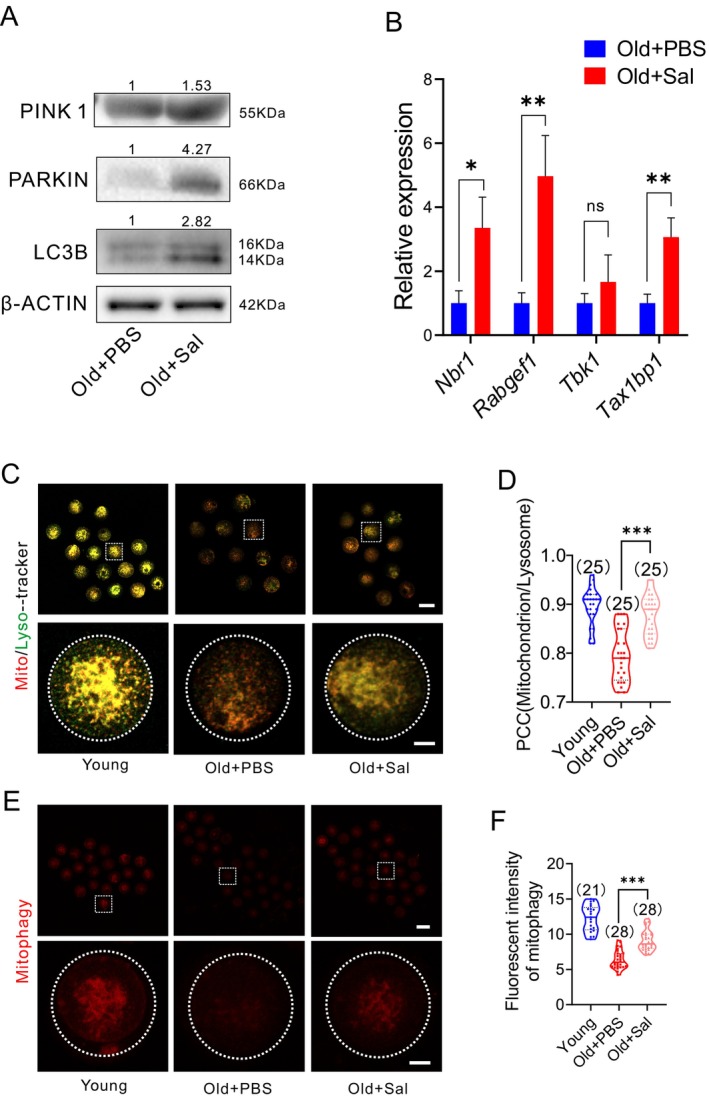
Salidroside (Sal) treatment improves mitophagy in oocytes from reproductively old mice. (A) Mitophagy markers demonstrate increased expression in GV oocytes of in vivo Sal‐treated mice, Old + PBS (*n* = 100), and Old + Sal (*n* = 100) treatment. (B) mRNA levels of mitophagy‐promoting genes in MII oocytes after Sal treatment during IVM. Old + PBS (*n* = 5 mice) and Old + Sal (*n* = 5 mice). (C) Representative images of mito‐tracker and lyso‐tracker staining of oocytes after in vitro maturation. Scale bar: 80 and 20 μm. (D) Pearson correlation coefficient shows the co‐localization of mitochondria and lysosomes. (E) Representative images of mitophagy dye staining of oocytes after in vitro maturation. Scale bar: 80 and 20 μm. (F) The fluorescence intensity of the dye indicative of mitophagy was measured in MII oocytes. *: *P* < 0.05; **: *P* < 0.01; ***: *P* < 0.001. The bar graph displays the mean ± SEM of the results obtained from at least three mice. The truncated violin plots depict the median, quantiles and individual data points. Numbers in parentheses represent the number of oocytes. Student's t‐test and one‐way ANOVA were used for statistical analysis.

To ascertain the effect of salidroside supplementation on mitophagy in aged oocytes, we first analyzed the protein levels of mitophagy‐related genes (PINK1, PARKIN, and LC3B). GV oocytes collected after in vivo salidroside treatment demonstrated increased protein levels of mitophagy‐related genes, PINK1, PARKIN, and LC3B, compared to age‐matched reproductively old controls (Figure 6A). We then assessed the expression of mitophagy‐promoting genes—*Nbr1, Rabgef1*, *Tbk1*, and *Tax1bp1*—using RT‐qPCR. The mRNA levels of *Nbr1*, *Rabgef1*, and *Tax1bp1* increased significantly in salidroside‐treated oocytes (Figure 6B). These results provide further evidence that salidroside upregulates the expression of mitophagy‐promoting genes in oocytes. Salidroside treatment also increased mitochondria‐lysosome co‐localization and significantly increased the levels of mitophagy‐associated fluorescent signal, which is detected using mitochondria‐selective pH‐sensitive fluorescent dye, in reproductively old mice oocytes suggesting improved mitophagy (Figure 6C–F).

## 4 Discussion

**FIGURE 7:**
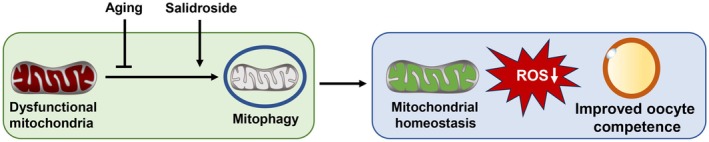
Salidroside improves oocytes competence by enhancing mitophagy. Aging impairs mitophagy in oocytes, which leads to mitochondrial dysfunction. Salidroside restores mitochondrial homeostasis, reduces ROS levels, and improves oocyte developmental competence.

Progressive depletion of the ovarian follicle reserve and a decline in oocyte quality with age results in increased infertility and miscarriage rates, and menopause (Faddy 2000; Y. Miao et al. 2020; Yamamoto et al. 2010). Understanding the mechanisms of and developing therapeutic strategies for reproductive aging has implications for fertility and women's overall health given that menopause is associated with adverse health outcomes (Davis et al. 2023). In this study, we investigated whether salidroside supplementation improves the oocyte development potential of reproductively old mice. Our data revealed that salidroside supplementation improves the folliculogenesis, fertility and maturation, fertilization, and subsequent embryonic development potential of oocytes from reproductively old mice. Salidroside improved spindle/chromosome alignment, mitochondrial membrane potential, mitochondria, and cortical granule distribution, and reduced ROS levels in the oocytes of reproductively old mice. Our transcriptomic and proteomic analyses point to enhanced mitophagy as a mechanism of salidroside action in oocytes (Figure 7).

A recent study reported that the addition of salidroside to the culture medium of postovulatory oocytes could delay their aging, maintain quality, and improve developmental potential in mice (K. Liu et al. 2024). Similarly, salidroside supplementation improved in vitro maturation and subsequent early embryonic development of porcine oocytes (J. Li et al. 2022; Shi et al. 2023). Our findings are consistent with these studies, expand on the in vitro effects of salidroside in oocytes, and additionally demonstrate the beneficial effects of salidroside on oocyte quality with reproductive aging utilizing in vivo models. We also provide a mechanistic insight into the salidroside action and suggest that enhanced mitophagy mediates its effects on the oocytes.

Reproductive aging impairs both nuclear and cytoplasmic oocyte maturation (Y. Miao et al. 2020; Y. L. Miao et al. 2009; Wu et al. 2022). Mitochondrial dysfunction is one of the several potential mechanisms contributing to ovarian aging (Ben‐Meir et al. 2015; J. Liu et al. 2012; T. Wang et al. 2017). Salidroside supplementation appears to improve both oocyte maturation and mitochondria function by increasing polar body extrusion rates, spindle/chromosome alignment on meiotic spindles, restoring cortical granule and mitochondria distribution, and enhancing ROS scavenging capacity and mitochondria function as evidenced by increased mitochondria membrane potential and ATP levels.

Our transcriptomic and micro‐proteomic analysis indicated that autophagy and mitophagy are the main pathways by which salidroside recovers oocyte quality in aged mice. Further investigations validated that exogenous salidroside increased mitophagy to restore oocyte quality in reproductively old mice. Our results are consistent with the previously reported mitophagy‐enhancing role of salidroside in the nervous system (R. Li and Chen 2019; Zhao et al. 2024). Moreover, increased mitophagy appears to be the predominant mechanism of action of spermidine to improve oocyte quality in reproductively old mice (Y. Zhang et al. 2023a), further suggesting this mechanism as a potential target for anti‐reproductive aging interventions.

In summary, our studies demonstrated that salidroside improves the quality and developmental competence of oocytes from reproductively old mice by promoting mitophagy. These findings provide a novel theoretical basis for the application of salidroside to improve oocyte quality in women with advanced reproductive age in the context of natural fertility or assisted reproduction.

## Author Contributions

T.W. conceived the original idea. T.W. and E.B. designed the experiments. J.G., R.H., H.W., and C.G. carried out the experiments. J.G., E.B., and T.W. analyzed and interpreted the data. J.G., R.H., E.B., and T.W. wrote the manuscript. R.H., K.L., W.S.B.Y., E.B., and T.W. provided critical discussion, reviewed, and revised the manuscript. All authors provided final approval of the manuscript before submission.

## Conflicts of Interest

The authors declare no conflicts of interest.

## Supporting information


**Figure S1.** Plasma concentration of salidroside after intraperitoneal administration in C57BL/6 female mice (*n* = 3).
**Figure S2.** mRNA levels of mitochondrial fusion and fission genes in MII oocytes after salidroside treatment during IVM. Old + PBS (*n* = 5 mice) and Old + Sal (*n* = 5 mice). *: *p* < 0.05; **: *p* < 0.01. The results represent mean ± SEM. Student’s *t*‐test was used for statistical analysis.
**Figure S3.** Transcriptomic analysis of MII oocytes after in vivo salidroside treatment (A) Volcano plot displaying the differentially expressed genes (DEGs) in MII oocytes between Old + PBS and Old + Sal groups. (B) Venn diagram of differentially expressed genes between GV oocytes and MII oocytes comparison groups. (C) KEGG enrichment analysis of DEGs in Old + Sal compared with Old + PBS MII oocytes. (D) GO enrichment analysis of DEGs in Old + Sal compared to Old + PBS MII oocytes.

**Table S1.** The list of primers used for quantitative RT‐PCR.

**Table S2.** mRNA levels in GV oocytes after salidroside treatment.

**Table S3.** mRNA levels in MII oocytes after salidroside treatment.

**Table S4.** Protein levels in GV oocytes after salidroside treatment.

**Data S1.** Materials and methods.

## Data Availability

All data generated in this study are available upon request from the corresponding authors.
