# Supplementary figures and images for: Salidroside Improves Oocyte Competence of Reproductively Old Mice by Enhancing Mitophagy
Source: Aging Cell. 2025 Jan 9;24(5):e14475. doi: 10.1111/acel.14475 (PMC12073897; doi:10.1111/acel.14475)

# Figures S1

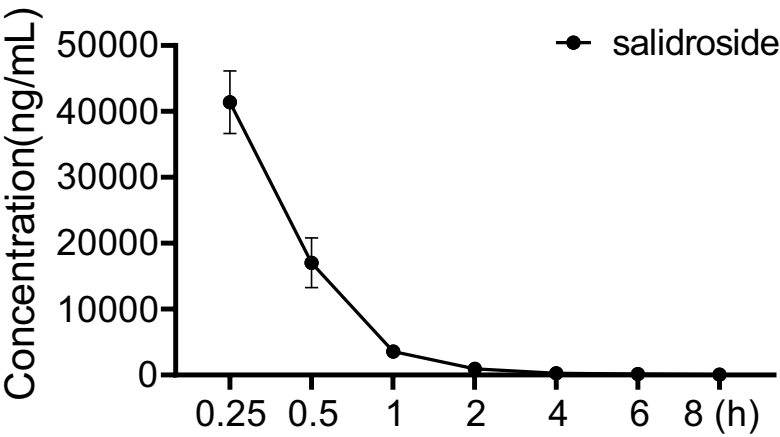

Figures S2

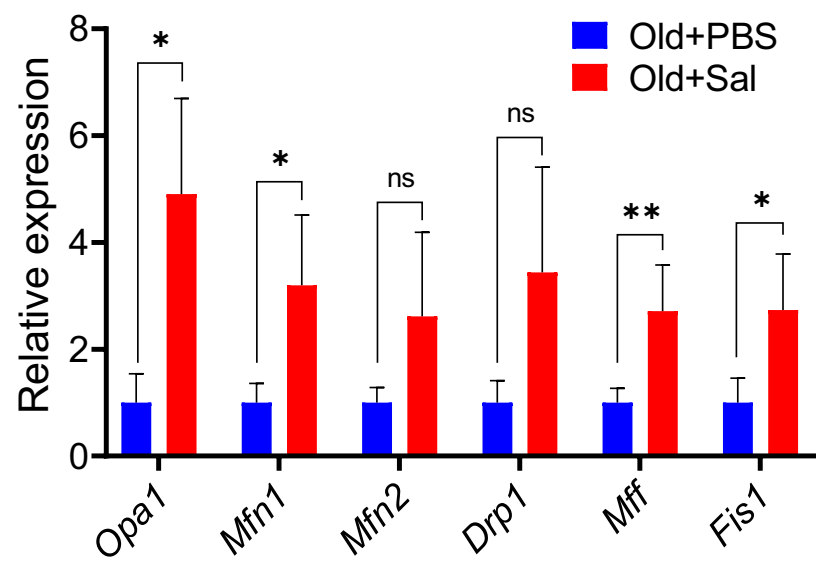

# Figures S3

A

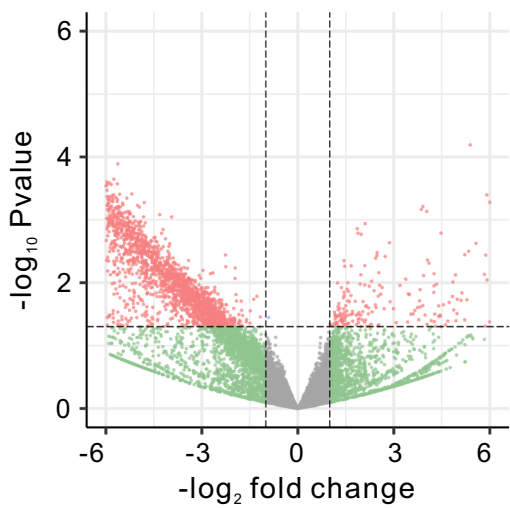

B

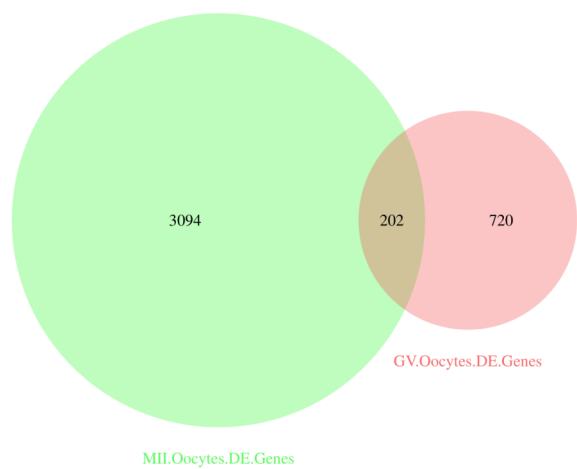

C

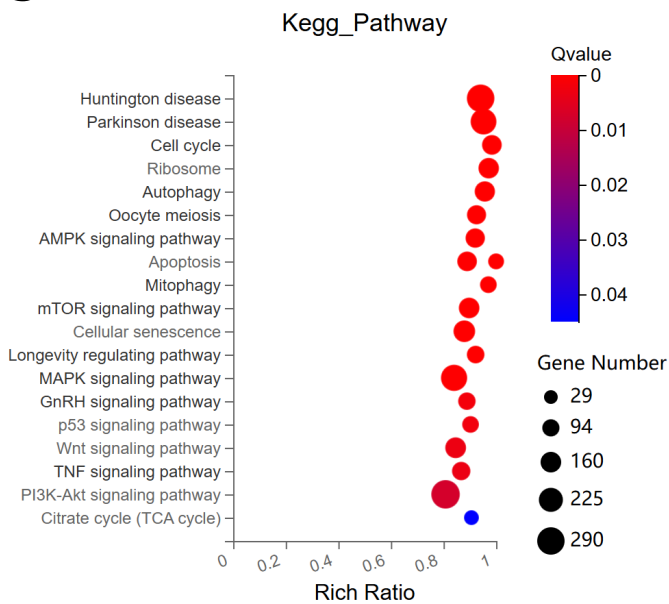

D

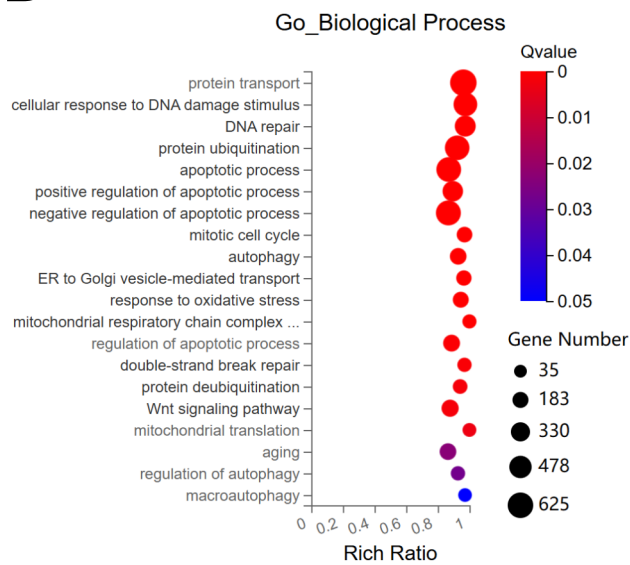

Supplement: Supplementary file 1 — Figure S1. Plasma concentration of salidroside after intraperitoneal administration in C57BL/6 female mice (n = 3). Figure S2. mRNA levels of mitochondrial fusion and fission genes in MII oocytes after salidroside treatment during IVM. Old + PBS (n = 5 mice) and Old + Sal (n = 5 mice). *: p < 0.05; **: p < 0.01. The results represent mean ± SEM. Student’s t‐test was used for statistical analysis. Figure S3. Transcriptomic analysis of MII oocytes after in vivo salidroside treatment (A) Volcano plot displaying the differentially expressed genes (DEGs) in MII oocytes between Old + PBS and Old + Sal groups. (B) Venn diagram of differentially expressed genes between GV oocytes and MII oocytes comparison groups. (C) KEGG enrichment analysis of DEGs in Old + Sal compared with Old + PBS MII oocytes. (D) GO enrichment analysis of DEGs in Old + Sal compared to Old + PBS MII oocytes. [file ACEL-24-e14475-s006.pdf]
