# Supplementary material for: Salidroside Improves Oocyte Competence of Reproductively Old Mice by Enhancing Mitophagy
Source: Aging Cell. 2025 Jan 9;24(5):e14475. doi: 10.1111/acel.14475 (PMC12073897; doi:10.1111/acel.14475)
Supplement: Supplementary file 2 — Table S1. The list of primers used for quantitative RT‐PCR. [file ACEL-24-e14475-s005.docx]

**Table S1. The list of primers used for quantitative RT-PCR.**

|  | **Primer sequences** |
| --- | --- |
| Opa1 Forward primer | 5′-CGACTTTGCCGAGGATAGCTT-3’ |
| Opa1 Reverse primer | 5′-CGTTGTGAACACACTGCTCTTG-3’ |
| Fis1 Forward primer | 5′-TGTCAAGAGCACGCAATTTG-3’ |
| Fis1 Reverse primer | 5′-CCTCGCACATACTTTAGAGCCTT-3’ |
| Drp1 Forward primer | 5′-CAAGGTTTTCTCGCCCAACG-3’ |
| Drp1 Reverse primer | 5′-CTGCCCTTACCATCTGGATCTA-3’ |
| Mfn2 Forward primer | 5′-GCTCCTGAAGGATGACCTCG-3’ |
| Mfn2 Reverse primer | 5′-CGTCTGCATCAGCGTGGACTC-3’ |
| Mfn1 Forward primer | 5′-CTGCTTCCTGAGTGTCGAGG-3’ |
| Mfn1 Reverse primer | 5′-ATGCACAAGACAGCCAGCTT-3’ |
| Mff Forward primer | 5′-CAGTTGGCAGGCTAAAAAGAGA-3’ |
| Mff Reverse primer | 5′-GCCCTACGAGTAGAAGACTGG-3’ |
| Gapdh Forward primer | 5′-TGGATTTGGACGCATTGGTC-3’ |
| Gapdh Reverse primer | 5′-TTTGCACTGGTACGTGTTGAT-3’ |
| Nbr1 Forward primer | 5′-GGAAATCAGCTACAGATGCAAGT-3’ |
| Nbr1 Reverse primer | 5′-ATCCCAAGACTCTCACCAGTG-3’ |
| Rabgef1 Forward primer | 5′-ATGAGCCTGAAGTCCGAACG-3’ |
| Rabgef1 Reverse primer | 5′-GCCTTGTGGTACTCCTCCCT-3’ |
| Tax1bp1 Forward primer | 5′-TGCACACTTGGAGTGCCATTA-3’ |
| Tax1bp1 Reverse primer | 5′-TGTTCAGGCATAGGAGACCATAA-3’ |
| Tbk1 Forward primer | 5′-ACTGGTGATCTCTATGCTGTCA-3’ |
| Tbk1 Reverse primer | 5′-TTCTGGAAGTCCATACGCATTG-3’ |
