# Supplementary material for: Salidroside Improves Oocyte Competence of Reproductively Old Mice by Enhancing Mitophagy
Source: Aging Cell. 2025 Jan 9;24(5):e14475. doi: 10.1111/acel.14475 (PMC12073897; doi:10.1111/acel.14475)
Supplement: Supplementary file 6 — Data S1. Materials and methods. [file ACEL-24-e14475-s001.docx]

**Materials and methods**

*Animals and in vivo treatment*

Mice were of the C57BL/6J background and were kept in the animal facilities at Peking University Shenzhen Hospital. The young (8-week-old) and reproductively old (10-month-old) female mice were kept at controlled conditions of temperature (23–25℃) and illumination (12 h light-dark cycle), and had free access to food and water throughout the period of the study. We chose 10-month-old timepoint because this represents a period of significant reproductive decline, similar to humans beyond mid-thirties (Cao et al., 2020; Nemerovsky et al., 2022). Aged mice were intraperitoneally injected daily with salidroside (SAL) (75 mg/kg body weight per day) or the equivalent volume of PBS for 14 consecutive days. The mice were housed in a 12-hour light-dark cycle with free access to water and food. All experimental protocols were approved by the ethics committee of the University of Hong Kong-Shenzhen Hospital.

*Measurement of salidroside levels*

Salidroside concentration in serum samples was detected using liquid chromatography-mass spectrometry. Liquid – liquid extraction (LLE) was used for sample preparations. 5 μL of supernatant was injected into the LC–MS/MS system for the analysis. The standard working solutions for salidroside were serially diluted with methanol to 20, 50, 200, 500, 2000, 5000, 20000 and 50000 ng/mL. Chromatogram collection and integration were processed by Xcalibur 3.0 (Thermo) and linear regression was performed using weighted coefficients 1/X^2^.

*Breeding trial*

Aged control and salidroside treated female mice (10-month-old, n=6 for each group) were housed continuously with WT (C57BL/6J) male mice for 24 weeks. The number of litters and pups were recorded for fertility analysis.

*Tissue collection and histological analysis*

The female mice were euthanized by cervical dislocation. The ovaries were immediately collected and fixed in 4% (w/v) paraformaldehyde with phosphate-buffered saline (PBS, Cat. C10010500BT, Thermo Fisher Scientific, MA, USA) overnight before dehydration, paraffin embedding, sectioning at 8 μm thickness and mounting on glass slides. The samples were then deparaffinized, stained with hematoxylin solution for 90 sec, washed three times with ddH2O, mounted with neutral balsam (Cat. G8590, Solarbio, Beijing, China), and imaged under a light microscope. The follicles at different developmental stages were counted, when the oocyte nucleus was visible, in every section to compare the numbers between groups. Primordial, primary, secondary, antral and atretic follicles were classified as previously described (Kerr et al., 2006; Myers, Britt, Wreford, Ebling, & Kerr, 2004).

*Oocyte collection*

Mouse oocytes were collected using previously described protocol (G. Li et al., 2023), starting with 10 IU PMSG (Cat. P9970-1000 Solarbio, Beijing, China) injected intraperitoneally to stimulate follicular development and subsequent collection of different types of samples at different time periods: (1) 44 hours after PMSG injection, the ovaries were collected and placed into M2 medium (Sigma, MO, USA). A disposable syringe was used to disrupt antral follicles. Cumulus cells were removed from collected cumulus-oocyte complexes (COCs) using a 75 μm pipette to obtain Germinal vesicle (GV) oocytes; (2) 48 hours after PMSG injection, an additional 10 IU of hCG (Cat.NB1122, NSHF, Ningbo, China) was injected to induce oocyte maturation and ovulation, and COCs were obtained from the fallopian tubes 14-16 hours after hCG injection. COCs were briefly placed into M2 medium containing 1 mg/mL hyaluronidase (Cat. H3506, Sigma, MO, USA) with gentle pipetting to remove cumulus cells, and metaphase II (MII) oocytes were obtained.

*In vitro fertilization (IVF) and embryo culture*

Caudae epididymides from 12-week-old male mice were lanced in a dish of HTF (Cat. M1135, AibeiBio, Nanjing, China) medium under mineral oil to release sperm, followed by capacitation for 1 h (37℃, 5% CO2). Sperm was then added to ovulated oocytes at a concentration of 4x10^5^/mL in 100 μL HTF for 5 h at 37℃,5% CO2. The presence of two pronuclei was scored as successful fertilization. The embryos were cultured in a 3 cm culture dish, in a drop containing 100 μL KSOM (Cat. MR-101, Sigma, MO, USA) under 3 mL mineral oil at 37℃, 5% CO2 atmosphere.

*In vitro maturation (IVM) of in vitro salidroside treatment and parthenogenetic activation (PA)*

GV oocytes were cultured in M16 medium (Cat. M7292, Sigma, MO, USA) at 37℃ in an atmosphere of 5% CO2 for 12 h. SAL was added to the IVM culture medium to achieve 25 μM concentration. The mature oocytes were transferred to CZB medium containing 10 mM/L SrCl_2_ (Cat. 255521, Sigma, MO, USA) and cytochalasin B (Cat. HY-16928, MedChemExpress, Shanghai China) (5 ug/ml) and cultured for 6 hours. The presence of two pronuclei was scored as successful activation. The embryos were cultured in a 3 cm culture dish, in a drop containing 100 μL KSOM under 3 mL mineral oil at 37℃, 5% CO2 atmosphere.

*Oocyte immunofluorescence*

Oocyte immunofluorescence was conducted using standard protocols (Wang et al., 2018). Oocytes were fixed in 4% (w/v) paraformaldehyde in PBS for 30 min, and permeabilized in 0.5% Triton X-100 for 5 min. Then, oocytes were incubated with 2 μg/mL anti-α-Tubulin (Cat.16-232, Millipore, Billerica, MA, USA) for 1 h, washed three times for 5 min in PBS and stained with 4’,6-diamidino-2-phenylindole (Cat#G1012-10ML DAPI, Servicebio Wuhan China), prior to being examined via a Zeiss LSM 900 confocal microscope, using excitation at 488 nm and emission at 530 nm (tubulin) and excitation at 350 nm and emission at 470 nm (DAPI).

*Evaluation of mitochondrial morphology*

Oocytes were incubated in M2 medium containing 500 nM MitoTracker™ Red CMXRos (Cat. M7512, Invitrogen, MA, USA) for 30 min at 37 ºC in a dark environment with 5% CO2 in air. After washing three times with fresh M2 medium (2 min each), oocytes were mounted on non-fluorescent glass slides for imaging. The images were captured by Zeiss LSM 900 confocal microscope. Oocytes with irregularly aggregated mitochondria in the ooplasm were marked as having abnormal mitochondria distribution, and this was quantified for all groups.

*Quantitative Reverse-Transcription Polymerase Chain Reaction*

Total RNA was extracted from 5 GV stage oocytes through VAHTS RNA Clean Beads (Cat. N412-01, Vazyme, Nanjing, China). The extracted RNA was reverse transcribed using the HiScript III RT SuperMix for qPCR (+gDNA wiper) (Cat. R323-01, Vazyme, Nanjing, China) according to the manufacturer’s instructions. Target genes and the housekeeping gene *Gapdh* were quantified using real-time qPCR with Taq Pro Universal SYBR qPCR Master Mix (Cat. Q712-02, Vazyme, Nanjing, China), and an Applied Biosystems 7500 Real-time PCR system (Cat. 4351107, Applied Biosystems™, Thermo Fisher Scientific, MA, United States). The following program was used: activation at 95°C for 3 min (1 cycle) and then 40 cycles of denaturation at 95°C for 15 s and annealing/extension at 60°C for 30 s. Relative mRNA levels were normalized to *Gapdh* and quantified using the 2−ΔΔCt method. See Table S1 for the list of primers.

*Assessment of mitochondria and lysosomes co-localization*

Oocytes were incubated in M2 medium containing 500 nM MitoTracker™ Red CMXRos (Cat. M7512, Invitrogen, MA, USA) for 30 min at 37 ºC in a dark environment with 5% CO2 in air. After washing three times with fresh M2 medium (2 min each), oocytes were treated with 500 nM Lyso-Tracker Green (Cat# C1047S, Beyotime Shanghai China) using the same protocol. After treatment, oocytes were mounted on non-fluorescent glass slides for imaging. The images were captured by Zeiss LSM 900 confocal microscope. The Pearson correlation was analyzed by Image J software.

*Mitochondrial autophagosome detection*

Mitochondrial autophagosomes were detected using the Mitophagy detection kit (Cat. MD01, Dojindo, Japan). Mitophagy dye emits weak fluorescence in intact mitochondria. When mitophagy is induced, the damaged mitochondria fuse to the lysosomes, and marked fluorescent signal is emitted (Iwashita et al., 2017). Oocytes were incubated with a 100 nmol/l mitophagy dye at 37°C for 30 min. Zeiss LSM 900 confocal microscope was used to capture the images using excitation at 530 nm and emission at 700 nm.

*Quantification of mtDNA copy numbers in oocytes*

The mtDNA copy number in oocytes was quantified according to previously established methods (Wang et al., 2018), pUC57-Cox3 plasmid (IGE, Guangzhou, China) DNA was quantified using NanoDrop 2000 spectrophotometer (Thermo Scientific, MA, USA). A standard curve from 10^8^ to 10^1^ plasmid molecules was generated by serial 10-fold dilutions. Single MII oocyte was lysed in 10 μL lysis solution (50 mM KCl,10 mM pH 9.0 Tris-HCl, 0.1% Triton X-100 and 0.4 mg/ml proteinase K) before incubating at 55ºC for 2 hours. Proteinase K was denatured subsequently by heating at 95ºC for 10 min and the mix was then used directly for downstream PCR in triplicates for each group. Each 10 μL reaction contained 5 μL of Taq Pro Universal SYBR qPCR Master Mix (Cat. Q712-02, Vazyme, Nanjing, China), 0.3 μM primers, and 3 μL oocyte DNA. The mtDNA copy number of each oocyte was then extrapolated from the standard curve.

*Determination of Reactive oxygen species (ROS) levels*

6-carboxy-2', 7’-dichlorodihydrofluorescein diacetate (carboxy-H2DCFDA; Cat. C-400, Life Technologies, ThermoFisher Scientific, MA, USA) was used to asses ROS levels in mouse oocytes using previously established protocol (Wang et al., 2018). MII oocytes were pre-treated with M2 medium containing 10 mM H2O2 for 5 min to induce ROS generation. They were then washed and incubated with 30 µM H2DCFDA in M2 medium for 20 min. Oocytes were washed 3 times with M2 medium and then immediately imaged by Zeiss LSM 900 confocal microscope. Image J software was used to quantify the total fluorescence intensity per oocyte.

*Evaluation of oocyte mitochondrial membrane potential*

Mitochondrial membrane potential was evaluated with JC-1 probe (Cat. T3168, Invitrogen, MA, USA). In brief, oocytes were incubated in M2 medium containing 2 μg/mL of JC-1 probe for 30 min at 37ºC in the dark and then washed 3 times (3 mins each) with M2 medium. These oocytes were immediately imaged under the Zeiss LSM 900 confocal microscope. JC-1 dye exhibits a potential-dependent accumulation in mitochondria as indicated by an emission shift of fluorescence from green (~529 nm) to red (~590 nm). Thus, mitochondrial depolarization is indicated by the red/green fluorescence intensity ratio.

*Quantification of ATP content*

The ATP content of individual oocytes was determined using the ATP Bioluminescent Somatic Cell Assay Kit (Sigma, MO, USA). Oocytes were lysed in 100 µL of somatic ATP release reagent. 100 μL of ATP mix working solution (diluted 1:25 from ATP Assay Mix stock solution) was added and mixed with the samples before loading to the 96-well plate and incubation at room temperature for 3-5 min to allow endogenous ATP hydrolysis. The amount of emitted light was immediately measured with a BioTek luminometer (Cat. SYNERGY H1, BioTek, Vermont, USA). Background luminescence was subtracted from all readings. ATP in individual oocyte samples was quantified based on generated standard curve in the range of 2.5–500 fmol/100 μL.

*Cortical Granule Staining*

*In vitro*-matured oocytes washed three times in PBS containing 0.4% BSA, and then fixed with 4% paraformaldehyde for 30 min at room temperature. The oocytes were blocked by washing them three times in a solution of 0.3% BSA and 100 mM glycine (Cat. G8790, Sigma, MO, USA). Then, they were permeabilized with 0.1% Triton X-100 for 5 min and incubated in 20 μg/mL fluorescein isothiocyanate (FITC)-labeled lens culinaris agglutinin (LCA) (Invitrogen, Grand Island, NY, USA). Finally, the oocytes were mounted on a slide in a 10-μL drop of glycerol and observed using an Zeiss LSM 900 confocal microscope at 515 nm for fluorescent CGs.

*Single-cell RNA sequencing*

Transcriptomic analysis of oocytes was carried out using a protocol for single cell RNA-Seq. In brief, 3 samples were collected for each group (3 oocytes per sample) in the lysis buffer that contains RNase inhibitors. Reverse transcription with oligo dT was used for to generate cDNA. Then cDNA was amplified by PCR, and the libraries were constructed after purification of amplified products, including DNA fragmentation, end repair, adding poly(A) and joint, PCR amplification and library quality control. The constructed library was sequenced with the Illumina platform. The sequencing strategy was PE150. The original down sequence (Raw Reads) obtained from Hiseq sequencing was completed through the process of removing low-quality sequences and connector pollution. High-quality sequences (clean reads) were obtained, and all subsequent analysis was based on clean reads.

*Protein Extraction and Western Blotting Analysis*

One hundred GV oocytes were collected from female C57BL/6 mice and suspended in the lysis buffer [50mM HEPES-KOH (pH 7.5), 100mM KCl, 2mM EDTA, 10% glycerol, 0.1% NP-40, 10mM NaF, 0.25mM Na3VO4, and 50mM ß-glycerophosphate] supplemented with complete protease inhibitor (Cat. 04693116001, Roche, Basel, Switzerland). The samples were homogenized and centrifuged at 20,000 g for 20min at 4°C, after which the supernatant was retained for western blotting analysis (Guo et al., 2023). The proteins in each sample were separated using 8–16% Bis-Tris gels (Cat. M00659, SurePAGE™, GenScript, Nanjing, China) and a mini protein electrophoresis system (Cat. 1658034, BIO-RAD, CA, USA) following the manufacturer’s instructions. The protein bands were then transferred to polyvinylidene fluoride (PVDF) membranes (Cat. IPVH00010, Immobilon, Millipore, MA, USA) via a Mini Trans-Blot Electrophoretic Transfer Cell (Cat. 1703930, BIO-RAD, CA, USA). The immunoreactive bands were detected and analyzed with a Bio-Rad ChemiDoc MP imaging System (Cat. 12003154, BIO-RAD, CA, USA) in conjunction with the Image Lab Software (Bio-Rad, CA, USA). The relative protein levels in each sample were normalized to ß-ACTIN to standardize the loading variations. The obtained images were analyzed by ImageJ software for gray value analysis.

*Microproteomics Analysis*

Microproteomic technology for low-input samples was used to investigate the proteomic changes in oocytes as previously described (H. Li et al., 2023). In brief, 2 samples were collected for each group (20 oocytes per sample) in the lysis buffer (7 M urea, 2 M thiourea, 20 mM Tris-HCl, pH 8.0) that contains protease inhibitors (Cat. 04693116001, Roche, Basel, Switzerland). Simultaneously, proteins from 8000 GV-stage oocytes from 3-week-old wild-type mice were extracted to construct the spectral library. Next, samples were separated by Thermo UltiMate 3000 UHPLC. First, the sample entered the trap column for enrichment and desalting and was then connected in series with a self-packed C18 column and separated at a flow rate of 500 nl/min. The peptides separated by the liquid phase were ionized by the nanoESI source and entered the high-resolution tandem mass spectrometer Orbitrap Fusion ™ Lumos ™ Tribrid™ (Thermo Fisher Scientific, San Jose, CA) for DDA (data-dependent acquisition) mode detection. The micro-sample data and the library sample data were used with MaxQuant mode to complete the identification. Then MaxQuant used the peptide peak intensity, peak area, and LC retention time related to micro-sample data and other information to perform the quality control on micro-samples. Finally, GO, COG, Pathway and other functional annotation analyses were performed on the data.

*Interaction network analysis of differentially expressed genes and proteins*

Interaction network analysis of differentially expressed genes and proteins was conducted using STRING (https://cn.string-db.org/). A differential co‑expression network was also constructed, and the submodules were screened using *MCODE* in Cytoscape software.

*Statistical analysis*

The bar or line graphs display the mean ± SEM of the results obtained from at least three mice. The truncated violin plots depict the median, quantiles and individual data points. Shapiro-Wilk test was performed normality analysis. Groups were compared with using t-test or one-way ANOVA test where appropriate. Statistically significant P-values (< 0.05, < 0.01 and < 0.001) were indicated by asterisks (*, ** and ***, respectively), while ‘ns’ represents not significantly different results. Graphs were generated using Microsoft Excel and GraphPad Prism 8. Figures were prepared with the CorelDraw version X8 (Corel Corp., Ottawa, ON, Canada).

Cao, Y., Zhao, H., Wang, Z., Zhang, C., Bian, Y., Liu, X., . . . Zhao, Y. (2020). Quercetin promotes in vitro maturation of oocytes from humans and aged mice. *Cell Death Dis, 11*(11), 965. doi:10.1038/s41419-020-03183-5

Guo, C., Xiao, Y., Gu, J., Zhao, P., Hu, Z., Zheng, J., . . . Wang, T. (2023). ClpP/ClpX deficiency impairs mitochondrial functions and mTORC1 signaling during spermatogenesis. *Commun Biol, 6*(1), 1012. doi:10.1038/s42003-023-05372-2

Iwashita, H., Torii, S., Nagahora, N., Ishiyama, M., Shioji, K., Sasamoto, K., . . . Okuma, K. (2017). Live Cell Imaging of Mitochondrial Autophagy with a Novel Fluorescent Small Molecule. *ACS Chem Biol, 12*(10), 2546-2551. doi:10.1021/acschembio.7b00647

Kerr, J. B., Duckett, R., Myers, M., Britt, K. L., Mladenovska, T., & Findlay, J. K. (2006). Quantification of healthy follicles in the neonatal and adult mouse ovary: evidence for maintenance of primordial follicle supply. *Reproduction, 132*(1), 95-109. doi:10.1530/rep.1.01128

Li, G., Gu, J., Zhou, X., Wu, T., Li, X., Hua, R., . . . Wang, T. (2023). Mitochondrial stress response gene Clpp deficiency impairs oocyte competence and deteriorate cyclophosphamide-induced ovarian damage in young mice. *Front Endocrinol (Lausanne), 14*, 1122012. doi:10.3389/fendo.2023.1122012

Li, H., Zhao, H., Yang, C., Su, R., Long, M., Liu, J., . . . Su, Y. Q. (2023). LSM14B is an Oocyte-Specific RNA-Binding Protein Indispensable for Maternal mRNA Metabolism and Oocyte Development in Mice. *Adv Sci (Weinh), 10*(18), e2300043. doi:10.1002/advs.202300043

Myers, M., Britt, K. L., Wreford, N. G., Ebling, F. J., & Kerr, J. B. (2004). Methods for quantifying follicular numbers within the mouse ovary. *Reproduction, 127*(5), 569-580. doi:10.1530/rep.1.00095

Nemerovsky, L., Bar-Joseph, H., Eldar-Boock, A., Tarabeih, R., Elmechaly, C., Ben-Ami, I., & Shalgi, R. (2022). The Role of PEDF in Reproductive Aging of the Ovary. *Int J Mol Sci, 23*(18). doi:10.3390/ijms231810359

Wang, T., Babayev, E., Jiang, Z., Li, G., Zhang, M., Esencan, E., . . . Seli, E. (2018). Mitochondrial unfolded protein response gene Clpp is required to maintain ovarian follicular reserve during aging, for oocyte competence, and development of pre-implantation embryos. *Aging Cell, 17*(4), e12784. doi:10.1111/acel.12784
